# Genome Sequences of Eight Prophages Isolated from *Lactococcus lactis* Dairy Strains

**DOI:** 10.1128/genomeA.00906-16

**Published:** 2016-11-10

**Authors:** Joana Oliveira, Jennifer Mahony, Gabriele Andrea Lugli, Laurens Hanemaaijer, Thijs Kouwen, Marco Ventura, Douwe van Sinderen

**Affiliations:** a School of Microbiology, University College Cork, Cork, Ireland; b Department of Life Sciences, Laboratory of Probiogenomics, University of Parma, Parma, Italy; c DSM Biotechnology Centre, Delft, The Netherlands; d APC Microbiome Institute, University College Cork, Cork, Ireland

## Abstract

P335 group phages represent the most divergent phage group infecting dairy *Lactococcus lactis* strains and have significant implications for the dairy processing industry. Here, we report the complete genome sequences of eight lactococcal prophages chemically induced from industrial lactococcal strains that propagate lytically on one of two laboratory strains.

## GENOME ANNOUNCEMENT

Phages infecting *Lactococcus lactis* have been classified into 10 taxonomic groups based on morphology and genetic content (1), with members of the 936, c2, and P335 phage groups being most frequently encountered in dairy production facilities (2). The P335 phages, whose members may exhibit a virulent or temperate life cycle (3), are among the most genetically diverse group of lactococcal phages (4). Recombination between the genomes of incoming virulent P335 phages and integrated prophages present in the *L. lactis* chromosome has been shown to result in adaptation to host-imposed infection hurdles, thus highlighting their plasticity and genomic mosaicism (5).

To assess the presence of temperate phages in our *L. lactis* collection, chemical inductions were conducted on 113 industrial lactococcal strains, using a treatment of 5 µg/ml mitomycin C (Sigma). The mitomycin C-treated cultures were filtered (0.45-µm filter; Sarstedt), and phage enumeration was conducted by a plaque assay (6) employing one of two identified hosts (*L. lactis* 3107 [7] or SMQ-86 [8]). Eight temperate phages were thus identified and propagated using either of these two hosts. Phage purification was achieved by cesium chloride (CsCl) gradient centrifugation (9), and genomic DNA was extracted as described before (10). Illumina MiSeq sequencing was performed, followed by genome assembly using MIRA version 4.0.2. Open reading frame (ORF) predictions were performed automatically by Zcurve (version 1.0) software (http://tubic.tju.edu.cn/Zcurve_V/). Functional annotation was performed using BLAST against sequences present in the NCBI and Pfam (http://pfam.xfam.org/) databases. Final annotations were revised using the HHpred tool (http://toolkit.tuebingen.mpg.de/hhpred) and manually edited using the Artemis software package (http://www.sanger.ac.uk/resources/software/artemis). These eight genomes were between 31 and 36 kb in length, with 35% G+C content. Since the exact genomic termini have not been determined experimentally, the integrase-coding genes were assumed as the first ORF in line with previously published P335 phage genomes. Between 48 and 52 ORFs were predicted for each phage, and four putative functional modules were identified: (1) the lysogenic cassette (negative strand), (2) the replication module, (3) the morphogenesis module, and (4) the lysis module; these are genomic features that are well described in lactococcal P335 phages (4, 11). Significant diversity was observed in the replication module and among the genetic loci encoding the tail terminal region components (data not shown) compared with previously sequenced P335 phages, such as Tuc2009, Q33, and phiLC3 (4, 11, 12). Currently, P335 phages are classified into one of four subgroups (4). Comparative genome analysis revealed that phages 86501, 50101, and 63301 exhibit a close relatedness to phage LC3, a member of the P335 subgroup III. For the remaining phages, 62501 appears to represent a new member of subgroup IV, 98201 belongs to subgroup II, while phages 28201, 50901, and 56701 appear to represent members of a new P335 subgroup.

### Accession number(s).

GenBank accession numbers are listed in Table 1.

**TABLE 1:** Accession numbers for the sequenced temperate bacteriophages

Temperate phage	Accession no.
28201	KX456206
50101	KX456207
50901	KX456208
56701	KX456209
62501	KX456210
63301	KX456211
86501	KX456212
98201	KX456213
